# Bayesian strategy selection identifies optimal solutions to complex problems using an example from GP prescribing

**DOI:** 10.1038/s41746-019-0205-y

**Published:** 2020-01-20

**Authors:** S. Allender, J. Hayward, S. Gupta, A. Sanigorski, S. Rana, H. Seward, S. Jacobs, S. Venkatesh

**Affiliations:** 1 Global Obesity Centre, Institute for Health Transformation, Deakin University, 1 Gheringhap St, Geelong, VIC 3221 Australia; 2 Applied Artificial Intelligence Institute, Deakin University, 75 Pigdons Rd, Waurn Ponds, VIC 3216 Australia; 3 School of Medicine, Deakin University, 1 Gheringhap St, Geelong, VIC 3221 Australia

**Keywords:** Lifestyle modification, Decision making

## Abstract

Complex health problems require multi-strategy, multi-target interventions. We present a method that uses machine learning techniques to choose optimal interventions from a set of possible interventions within a case study aiming to increase General Practitioner (GP) discussions of physical activity (PA) with their patients. Interventions were developed based on a causal loop diagram with 26 GPs across 13 clinics in Geelong, Australia. GPs prioritised eight from more than 80 potential interventions to increase GP discussion of PA with patients. Following a 2-week baseline, a multi-arm bandit algorithm was used to assign optimal strategies to GP clinics with the target outcome being GP PA discussion rates. The algorithm was updated weekly and the process iterated until the more promising strategies emerged (a duration of seven weeks). The top three performing strategies were continued for 3 weeks to improve the power of the hypothesis test of effectiveness for each strategy compared to baseline. GPs recorded a total of 11,176 conversations about PA. GPs identified 15 factors affecting GP PA discussion rates with patients including GP skills and awareness, fragmentation of care and fear of adverse outcomes. The two most effective strategies were correctly identified within seven weeks of the algorithm-based assignment of strategies. These were clinic reception staff providing PA information to patients at check in and PA screening questionnaires completed in the waiting room. This study demonstrates an efficient way to test and identify optimal strategies from multiple possible solutions.

## Introduction

The major global challenges to population health are characterised by their complexity, multiple relations of cause and effect, differing time scales and resistance to single programmatic intervention. Recent moves to address this complexity have looked to systems science with an emphasis on co-creation with key stakeholders to develop feasible and context relevant interventions.^1^ The next generation of health intervention needs to embrace complexity and create multiple changes at multiple levels of systems to be more effective. While promising, this approach will necessarily generate many possible alternate strategies that need to be evaluated.

Typical designs to test multiple interventions involve either multiple replications of separate single interventions against a control or some form of selection of optimal intervention by sequentially trialling each strategy across both control and intervention groups. Testing multiple different possible interventions requires large numbers of experimental conditions. For example, using a factorial design to compare three interventions would require eight separate experimental groups while testing nine alternate interventions would require 256 separate combinations of intervention and control.^2^

The use of modern machine learning methods may provide new ways of optimising strategy selection for health interventions while trialling multiple strategies simultaneously. The field of adaptive experimental optimisation^3,4^ has successfully utilised Bayesian methods for design of products and processes to test multiple possible problem solutions. For problems with a large set of discrete choices Bayesian multi-armed bandit algorithms have become popular due to their sample efficiency.^5^ A second machine learning technique, reinforcement learning, has been used in “just-in-time” adaptive intervention (JITAI) optimisation^6^ starting with individual user/patient data to select from a set of possible interventions.

The JITAI approach has been used with the goal of optimising treatment selection by individual whereas the MAB identifies strategies for application across groups, or populations of beneficiaries. While JITAI is sensitive to patient context and varies intervention over time with changing individual patient circumstances, (e.g. attitudes and beliefs etc.), the MAB enables assessment of change at the level of the setting where the goal is to influence behaviour across groups of people.

Simultaneously, methods are emerging to engage key stakeholders in understanding complexity, identifying priorities and defining multiple possible interventions to improve health. Participatory techniques have been used to engage stakeholders in developing causal loop diagrams^7^ that are in turn used to develop and prioritise multiple actions, form working groups and drive intervention design.^8^ This approach rests on the assumption that strong engagement with the key actors in a community will lead to multiple actions at multiple levels of a system. Many of these trials assume the more activities implemented the better.^9^

One example of the complexity indicated above can be seen in the general practitioner’s (GP) communication with patients about improving physical activity. Given that more than a quarter of the world’s population are insufficiently active to engender a health benefit,^10^ GPs have been seen as a powerful possible actor to initiate the conversation about improving this important health behaviour.^11,12^ For the GP the range of possible interventions becomes incredibly large ranging from individual genetic profiles, individual attitudes, knowledge and behaviours, family and organisational culture and environments through to diverse social determinants of health such as economic and social conditions.

Several examples demonstrate that health professionals’ interactions with patients provide a potentially beneficial site for behavioural intervention. A New Zealand based study^13^ demonstrated that GP provision of written and oral advice during patient consultations, supported by ongoing support from exercise specialists, resulted in significant increases in energy expenditure and recreational exercise and increases in quality of life compared with usual care 12 month post initial consultation. Subsequent editorials in the Lancet suggested “brief interventions” in GP consultations like mentioning behavioural change with referral as part of usual consultation has positive effects on modifiable health risks for up to a year after the consultation regardless of apparent readiness to change.^14^ The published trial literature has focussed primarily on exercise referral schemes (ERS) with overall mixed results. One systematic review^15^ reported no clear positive effect across seven RCTs of ERS on physical activity rates where comparison groups ranged from usual care, alternate physical activity referrals to counselling services. Patnode et al.^16^ reviewed 88 separate behavioural counselling trials for the primary prevention of cardiovascular disease in adults without known cardiovascular risk factors. Though they observed no overall effect on cardiovascular mortality or morbidity there was evidence of small though statistically significant improvements in intermediate disease markers at 6 and 12 months including blood pressure, cholesterol and healthy weight status and some evidence of impact on improved diet and physical activity. A third systematic review^17^ summarised trials to promote physical activity in the primary care setting, with interventions delivered by GPs, physiotherapists, health promotion and exercise specialists and comprising a range of ERS, motivational interviewing and counselling finding a small to medium positive effect on self-reported physical activity at 12 months.

Less than 20% of consultations include any discussion of PA^18^ and it is unclear how to increase the rates of GPs discussing PA with their patients. Where advice is given it is typically non-specific as, where studied, GPs report they do not feel they have adequate training or information to discuss PA with their patients,^19^ are not aware of referral options^20^ and see discussing PA as outside the core business of the GP practice.^21^

In this paper we report on a research project that aimed to study the following questions:

1. What are the best interventions to increase the rates of GPs discussing PA with their patients?
  - What are the main reasons for GPs low rates of discussing PA with their patients in a sample of GPs in Victoria, Australia?
  - What are the most feasible interventions that can be trialled to increase rates of GPs discussing PA with their patients?
  - What are the best intervention (s) to increase the rates of GPs discussing PA with their patients?
2. Is it possible to use modern machine learning methods to identify the optimal health intervention strategy in the shortest timeframe?

To address these questions, a multi-arm bandit algorithm was used to assign optimal strategies from a potential choice of eight interventions. This was implemented across 13 clinics involving 26 GPs. The two most effective strategies were correctly identified within seven weeks of commencing the algorithm-based assignment of strategies. This study demonstrates an efficient way to test and identify optimal strategies from multiple possible solutions.

## Results

### Reasons for GPs low rates of discussing PA with their patients

**Fig. 1:**
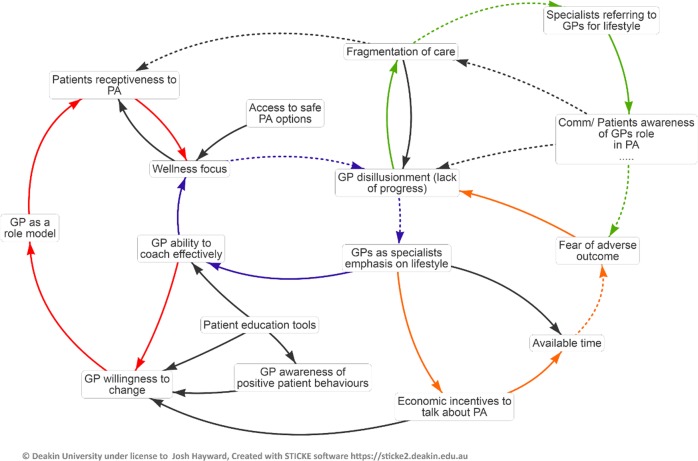
Causal loop diagram describing the factors affecting GPs rates of recommending PA to patients as a preventative measure. Labels denote a factor affecting PA recommendation as identified by participants, while arrows denote the causal relationships between factors. Solid arrows indicate direct relationships, while dotted arrows indicate inverse relationships. Feedback loops that captured important stories are highlighted in colour.

Participants identified 15 factors affecting GPs rates of recommending PA (Fig. 1). Most of the factors were located within four feedback loops that described issues that have traditionally stifled GPs willingness to discuss PA in the clinic. The four feedback loops were: “Disillusioned GPs not seeing themselves as lifestyle experts, and feeling unable to coach effectively” (Purple loop, centre), “GPs feeling they are a bad example that patients will not follow” (Red loop, left), “GPs referring out for PA advice, and public perception that GPs are not lifestyle experts” (Green loop, upper right), and “GPs not tackling PA for fear of adverse outcomes” (Orange loop, lower right). These stories aligned well with the previously reported barriers to GPs recommending PA discussed above^19–21^ and supported the subsequent conversation about strategies that might overcome these obstacles.

### Proposed interventions

Participants prioritised eight potential interventions to increase GPs recommending PA as a preventative health measure in Geelong:

1. Physical activity coaching mentors. Under this strategy GPs received coaching and mentoring on discussing PA with patients from an accredited exercise physiologist.
2. Physical activity recommendation tools. As part of a routine consultation, GPs briefly asked each patient about their level of PA. Based on this discussion the GPs recommended an appropriate level of PA using a “physical activity prescription,” guided participants through the completion of a SMART goal sheet relating to their PA objectives, and gave the patient a pamphlet outlining the Australian PA recommendations and associated health benefits.
3. Physical activity information given to patient by receptionist at check-in. Reception staff handed patients a small pack of information about physical activity, including printed copies of publicly available fliers from Australian health organisations (for example see heartfoundation.org.au/images/uploads/publications/I_can_be_active_today.pdf). The pack of information could then be used to prompt discussion in consultation with the patient.
4. Physical activity information given to patient by GP during consultation. GPs will hand patients a small pack of information about physical activity, including printed copies of publicly available fliers from Australian health organisations (for example see heartfoundation.org.au/images/uploads/publications/I_can_be_active_today.pdf). The pack of information could then be used to prompt discussion in consultation with the patient.
5. Posters promoting physical activity on display around the GPs clinic, and in consultation rooms. Reception staff placed publicly available posters from an Australian health organisation (livelighter.com.au/Assets/resource-vic/poster/AP1300_LL_Poster-A3_Stairs_FA_online_v2.pdf, livelighter.com.au/Assets/resource-vic/poster/AP1300_LL_Poster-A3_Walk-to-shops_FA_online_v2.pdf) around the clinic. During consultations, GPs could either begin a conversation around PA if the patient noticed the posters and asked a related question, otherwise they could start a conversation by asking patients if they noticed the posters as a talking point to begin a conversation.
6. Integration of physical activity information into Health Pathways. Health Pathways (westvicphn.com.au/health-professionals/healthpathways) is an on-line source of information on assessment, management and local referral options for GPs and other primary health care providers. Through partnership with local administrators of the system, information was added to help GPs connect quickly with information and resources relating to PA during the consultation, to support conversation with the patient.
7. Administration of a physical activity assessment completed by the patient in the waiting room. At check in, reception staff handed patients a questionnaire (assets.publishing.service.gov.uk/government/uploads/system/uploads/attachment_data/file/192450/GPPAQ_-_pdf_version.pdf) to complete while they sit in the waiting room. Discussion about PA could then be prompted either by the patient if they ask directly about the survey, or by the GP asking the patient about their results.
8. GP supported/recommended physical activity groups. For this strategy, GPs showed patients information available online about local opportunities to participate in social physical activity groups, centred around walking clubs in the local area.

### Best interventions to increase the rates of GPs discussing PA with their patients

**Fig. 2:**
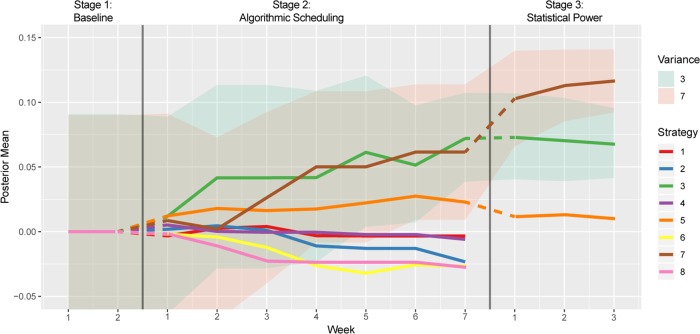
Model-based mean estimates for each strategy vs time. For clarity, the variance is only shown for the top two strategies. Stage 1: Weeks 1–2 no strategy is implemented. Stage 2: Weeks 1–7 the strategies are picked by the algorithm. Stage 3: Weeks 1–3 implements only the top three strategies determined at end of Stage 2 (3, 7, and 5) to improve the power of hypothesis testing (Strategy vs baseline).

Figure 2 shows the progression of each strategy’s mean effectiveness relative to the baseline over time. As the uncertainty around a strategy’s mean decreases, the mean will often flatten, with reduced expectation of change in the mean over subsequent trials. As example, five of the strategies reached a steady state mean after 3 weeks.

At the 1st week of Stage 2, the difference between all the strategies and the baseline appeared low; by week 2, strategy 3 and 5 started to emerge as promising strategies. By week 4 the algorithm had collected more data and strategy 7 emerged as an equal contender to strategy 3 outperforming strategy 5, which still seemed better than all the other strategies. This pattern remained the same with greater confidence emerging in the subsequent weeks. By week seven, strategy 3 and 7 were clearly identified as the best strategies, followed by strategy 5. Some strategies appeared to negatively affect discussion rates relative to baseline.

At the end of Stage 2, a hypothesis test using one-sided *t*-test was performed (see supplementary table 1). The decision to discontinue some of the strategies in Stage 3 was taken based on this hypothesis test and the posterior mean distribution of Thompson sampling. As seen from the supplementary table 3, strategies 7, 3 and 5 were more promising (based on their mean effect and *p* value) than the other strategies at this point. Further data collection from stage 3 ensured the statistical significance of strategies 7 and 3 being better than baseline at a significance level of 0.006 (= $\frac{{0.05}}{8}$, chosen based on Bonferroni correction due to involvement of 8 strategies).

**Fig. 3:**
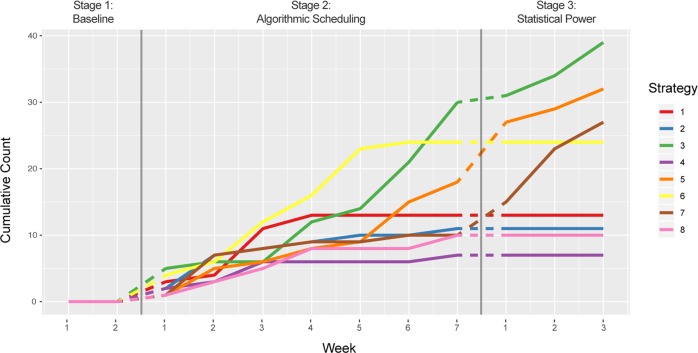
Cumulative number of trials conducted for each strategy vs time. Stage 1: Weeks 1–2 no strategy is implemented. Stage 2: Weeks 1–7 the strategies are picked by the algorithm. Stage 3: Weeks 1–3 implements only strategies top three strategies determined at end of Stage 2 (3, 7, 5) to improve the power of hypothesis test (Strategy vs baseline).

Figure 3 shows the cumulative number of trials applied for each strategy over time. The best performing strategies were typically allocated a larger number of trials with the exception of strategy 6, which was trialled more than expected because delayed data submission from some GPs led to higher uncertainty for its efficacy in the algorithm.

Strategies seven and three clearly emerge as the best strategies, with low standard error and *p*-values (Table 1). Mean and standard error of the reward distribution for each strategy were computed with a one-sided *t*-test at the end of the trial. The trial count is the cumulative count of number of trials for that strategy across all clinics at the termination of the trial.

**Table 1:** Final statistics for all strategies.

Strategy number	Mean difference (strategy-baseline)	Standard error	*P* Value	Trial Count
7	0.15	0.04	0.000	27
3	0.09	0.03	0.002	39
4	0.02	0.04	0.368	7
5	0.01	0.03	0.407	32
1	−0.01	0.04	0.651	13
2	−0.02	0.05	0.640	11
6	−0.03	0.03	0.863	24
8	−0.08	0.03	0.982	10

One sided *t*-test comparing estimates for mean estimate of difference in GP/patient consultations on PA

The strategies are sorted by descending difference in mean effects of strategy and baseline. The strategy 7 and 3 mean differences are statistically significant with *p*-values < 0.006

## Discussion

Using this machine learning approach two effective strategies were identified from the eight strategies prioritised by GPs intended to increase the number of discussions of PA with patients. These strategies, providing physical activity information to the patient at check-in and administration of a physical activity questionnaire at reception, were identified to be effective in increasing GP patient discussions of PA within 3 weeks of the algorithmic assignment. Ineffective strategies were identified within five weeks.

The algorithm tested multiple strategies at the same time exploiting effective strategies providing real time adaptation of the experimental condition based upon emergent learning about strategy effectiveness. Anecdotally the participating GPs enjoyed the process and the opportunity to participate in a new, low cost-efficient method of testing new approaches to increase GP prescribing of PA.

This approach allows all intervention options to be tested simultaneously and in real time where results are shared and analyzed weekly and actively used to direct the next interventions to be tested. Our case study meets the conditions of an adaptive intervention^22^ where decision points are weekly (advantage of timeliness) and tailored to a logic model informed by local evidence, decision rules, and intervention options. We worked with a group of GPs to capture their understanding of an existing problem, define possible solutions and test these in real time to find the best intervention options. A major strength of this approach is that it presented an optimal intervention mix within 2 months, allowing GPs to identify a productive course of action very quickly. These techniques also engaged those responsible for the implementation in the intervention design and so is more likely to actively consider and engage with potential limitations and facilitators of intervention success as part of the design.

A further strength is the inherent scalability of the method–software options to develop causal loop diagrams are readily available and able to be used after a short training period while the optimisation approach can be applied remotely. The machine learning approach confers the clear advantage of being able to test in real time and optimise as opposed to other intervention designs, like a factorial design, which would require 512 different experimental conditions to test and compare the range of intervention combinations and interactions.

Relative to factorial designs this case study has the limitation of not allocating equal budget to all strategies, which may be important if the goal is to estimate the efficacy of each strategy irrespective of whether their efficacy is small or large. As the Thompson sampling algorithm allocates the available budget in favour of well performing strategies over time, we did not maintain power to distinguish between the poorly performing strategies and the baseline after Stage 2.

The approach described in this paper is most promising where the interventions are clearly defined and readily acceptable by those who have to implement them, and the outcomes can be rapidly measured with a high degree of confidence. The approach is particularly appropriate where there is an engaged group of stakeholders interested in improving an existing problem (in this case GPs wishing to take a greater role in promoting PA), and the stakeholders are prepared to participate in defining the causes of the problem and potential intervention options. The approach cannot replace the clinical trial where there is a need to closely monitor adverse and unexpected consequences and where any emerging adverse outcomes might lead to trial cessation.

This model assumes that each effect of each strategy is constant over and should be used with caution if temporal trends in outcomes exist. A second assumption is that GPs are independent from each other (in this case both within and across clinics). Each of these assumptions were tested and confirmed (see Supplementary Figs. 1 and 2).

We also note that there is a small likelihood of the strategies dropped after Stage 2 being underestimated and thus coming out as better than the baseline if they were given more chances. However, we had our budgetary constraints in this study and therefore chose to concentrate on high-value strategies. Therefore, although the dropped strategies seemed to be ineffective, more study may be required to completely rule them out.

We have chosen Thompson sampling as the adaptive response strategy. This technique guarantees optimality asymptotically and thus in a finite-time scenario, other methods such as dynamic programming or Gittins indices based design may achieve better efficiency if the trial length is known a priori. An advantage of Thompson sampling algorithm however is that it allows incorporation of various types of complex prior knowledge (where available) about the strategies by deriving appropriate posterior distributions in traditional Bayesian frameworks.

Another aspect to be mindful of is that Thompson sampling trades between the type-1 error rate and statistical power, implying that to achieve a greater statistical power (relative to a same sample-size equal allocation trial), it is likely to inflate the type-1 error rate.^23^ By not trying the null strategy sufficiently, the type-1 error rate may exceed the acceptable threshold. Thus it is judicious to monitor the type-1 error rate and if it exceeds the significance level, adjustments may be required in the allocation algorithm. Recent work^24^ is starting to explore adaptive sampling approaches that can control the type-1 error rate, and further work is required to develop such techniques for a Thompson sampling setting.

We did not assess intervention fidelity, that is, whether the intervention was delivered as conceived and in accordance with the materials and instructions provided to participants. Given that the aims were to measure the effectiveness of the strategies in supporting GP-patient interactions where physical activity was discussed, failure to implement the strategies in consultations was ultimately what was tested, in essence assessing the utility of each strategy.

This case study has demonstrated an efficient process providing clear results in a short period of time. Further work should develop methods to understand interactions and sequencing of various and multiple combinations of interventions. Subsequent lines of investigation should also begin to consider the question of fidelity. Given this study demonstrates a process for supporting conversations about PA between GPs and patients, an important next step is to examine the quality and content of those conversations and their impact on measured patient PA.

While the reasons for low incidence of GP patient interaction about PA are complex, these interactions occur in a relatively stable setting with a clear, well-defined outcomes. Further work on other complex problems in similarly well-defined domains, or further investigations of the GP-patient relationship in expanded settings (i.e. with greater numbers of prioritised actions, or broader participant groups) represent exciting directions for future work.

The new frontier of public health is not what behaviours need to change, but rather how to change them. This study shows how new methods can be used to test and optimise implementation of intervention in multiple settings by engaging key actors in a system and moving quickly to the best set of interventions. For PA specifically, data suggested more than 80% of adults visit their GP at least once per year (www.moh.govt.nz/moh.nsf/ea6005dc347e7bd44c2566a40079ae6f/d7b3cf1eee94fefb4c25677c007ddf96). Rapid identification of appropriate strategies that increase the discussion of PA between GPs and their patients provides new potential avenues for the improvement of public health.

## Methods

### Context

This study was conducted in the city of Geelong, Victoria, Australia. The study involved a partnership between Deakin University, Active Geelong and the Western Victorian Primary Health Network (Westvic PHN). The former is a collective of civic leaders with the intent of increasing physical activity in the city of Geelong and the latter is a federally funded body charged with improving the quality and safety of patient care across Western Victoria.

### Development of interventions

Interventions were developed using techniques from participatory community-based system dynamics which deliberately set out to work with practitioners to understand complex problems and co-create potential solutions. Specifically, we used techniques from group model building^25^ to build a causal loop diagram (CLD) of GPs understanding of the causes and effects of low rates of GPs discussing PA with their patients during normal consultations. Participants were 15 GPs recruited through Westvic PHN who attended several workshops facilitated by a research team which had experience and training in the use of group model building. In the first workshop participants were provided with the evidence base on what is known about the effectiveness and current rates of GP PA prescription on patient PA rates and then asked to consider the seed question:

What factors affect GPs rates of recommendation of PA as a preventative health measure?

These factors were entered into causal mapping software and participants then identified which of the variables were connected in relationships of cause and effect and the direction and nature of the relationship (i.e. direct or inverse causal relationship). The mapping software supported the creation of a causal loop diagram from this information providing a grounded logic model of these GPs mental models for the rates of GP PA prescribing. In the second workshop, participants were asked to review and refine the initial model which was subsequently used to identify existing areas of action to increase PA prescription and where additional focus should be directed. Participants were then asked to consider what actions might be taken within this system to increase prescribing. This created 80 actions and these were subsequently prioritised according to participants understanding of the feasibility and likely impact of each intervention. In a third workshop, participants worked in small groups to provide detail on practical steps for implementing the prioritised actions.

### Study of the interventions

Thirteen clinics were recruited to trial the strategies for increasing the number of GP–patient conversations about PA. Across the 13 clinics, a total of 26 GPs participated in the study, recording a total of 11,176 conversations.

During the implementation phase of the study, intervention strategies were allocated to each GP weekly on a Monday and trialled from Tuesday to Friday. Where multiple participant GPs worked in the same clinic, they were allocated the same strategy to prevent cross-contamination. PA discussion data were recorded daily and returned to the research team after the last consultation of the week. Using the data obtained from all the clinics, the strategies for the next week were recommended for each clinic, with the implementation phase running for a total of 13 weeks (including 2 weeks of baseline data collection). All necessary materials and instructions for each of the intervention strategies were provided to the clinics ahead of time.

During implementation the machine learning algorithm was used to assign the strategies to the clinics until some strategies were identified as performing better than the baseline. This process lasted for seven weeks, before an additional 3 week period was dedicated to the strategies that emerged from the initial seven week window. This period was used to improve the power of the hypothesis test.

### Measures

The data collected for the study was prepared by GPs at the participating clinics. GPs would prepare a summary of their appointments over the week, noting against each appointment whether:

- Physical activity was discussed.
- Physical activity was not discussed.
- Physical activity was inappropriate in the context of the appointment (i.e. profound disability, or other equivalent medical considerations).

Baseline: in order to deal with variation between clinics (e.g. caused by different GPs, patient cohorts etc), each of the participating clinics collected 2 weeks of data without any of the proposed strategies being implemented. This “no action” data formed a baseline against, which the eight proposed strategies could be compared.

The baseline outcome for each clinic is the fraction of patient consultations (for all GPs) that touched on physical activity with no intervention applied in Weeks 1 and 2.

### Analysis—machine learning approach

The purpose of using the machine learning method algorithm was three-fold:

1. Identify the most promising strategy as quickly as possible.
2. Explore the variety of strategies enough to ensure a strategy is not unjustifiably overlooked.
3. Once a promising strategy (or strategies) emerges from the data, use the remaining trials available to maximise statistical power for these strategies.

**Fig. 4:**
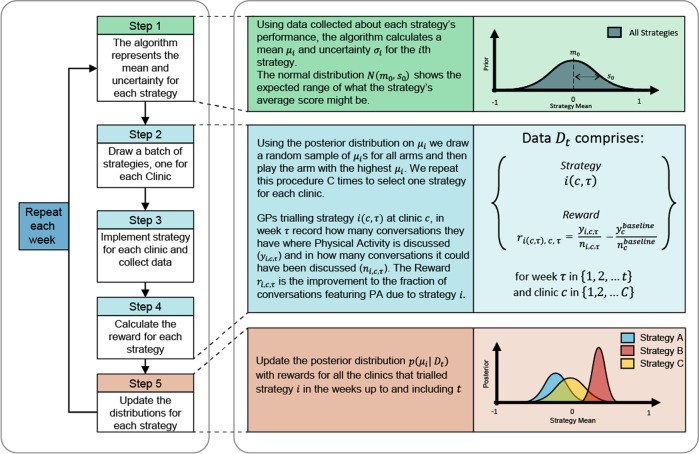
Architecture of the automated strategy selection. Left pane shows the 5 iterative steps of the proposed strategy optimisation. The right pane provides explanatory details.

Our overall architecture is shown in Fig. 4. The problem, from a machine learning perspective, is called a Multi-Arm Bandit.^26,27^ The name is inspired from the scene of a gambler (player) facing a series of slot machines (arms) who has to make the following decision: which machine should I play, and in what order so as to either maximise the total pay-off (reward) or identify the best machine in a minimum number of trials? The reward of each slot machine is assumed to be stochastic with an unknown mean at the start. Each time a slot machine is tried, a sample of its reward is observed, which helps in estimating the mean reward of the machine along with its epistemic uncertainty. While deciding which machine to try, the player needs to keep a balance between the two conflicting objectives—exploitation and exploration—focusing on already discovered slots (exploiting) with “promising” rewards to reach a higher confidence vs trying unexplored slots to find a slot with better reward. In our case, each intervention strategy is an arm of the multi-arm bandit.

In this work, we use a variant of multi-arm bandit algorithm called Thompson sampling. Thompson sampling estimates a probability distribution of the mean reward of each arm using any available observations. When making decision about which arm to pull next, it draws a sample of the mean reward for each arm from their respective probability distributions. The arm with the maximum random sample is chosen. A unique property of Thompson sampling is that it uses the full distribution of the arm means to balance exploration (also known as “learning”) and exploitation (also known as “earning”). Unlike other methods such as Upper Confidence Bound, it does not require the designer to specify the balance level between the exploration and exploitation. Theoretical behaviour of Thompson sampling has been recently analysed by several researchers^28,29^ and it has been shown to achieve the order optimal convergence rate asymptotically. In particular, since Thompson sampling is a randomised algorithm, it is common to analyse Bayesian cumulative regret *R*_*T*_ after *T* iterations, which has been shown to grow only sub-linearly in *T*. The convergence is guaranteed asymptotically as in the limit *T*→*∞*, the average regret $\frac{{R_T}}{T} \to 0$.

The algorithm begins by assuming a stochastic distribution of reward for each arm, to be initially non-informative—each is a Gaussian with a mean reward of 0 with a standard deviation of 0.3. This is because we expect the reward to lie in the interval [−1,1]. At each iteration, based on the exploitation-exploration trade-off policy and the current reward distributions of arms, an arm is chosen. We choose 13 arms (strategies), one for each clinic. For each clinic, the chosen strategy is implemented and used by all GPs in that clinic. It is important to note that a strategy may be played in more than one clinic and may also be repeated over time to reduce the uncertainty over its reward.

The outcome for each clinic for a particular strategy at the end of the intervention week is the fraction of the number of conversations (for all GPs) that included a discussion of physical activity, *y*, out of all the conversations where a discussion of physical activity could be relevant, *n*. Conversations deemed irrelevant included difficulties with a disability, patients who were infants, and patients with a drug use problem. The outcome of strategy *i* at clinic *c*, in week *τ* of the trial is thus given by (see Eq. (1)):

1 $$p_{i,c,\tau } = \frac{{y_{i,c,\tau }}}{{n_{i,c,\tau }}}$$

The reward for a strategy for a particular clinic in week *τ* is then computed as the difference between the outcome for the tested strategy $p_{i,c,\tau }$ and the outcome for the baseline strategy for that clinic, $p_c^{baseline}$ (see Eq. (2)):

2 $$r_{i,c,\tau } = p_{i,c,\tau } - p_c^{baseline}$$

Formally, the algorithm then proceeds as follows: Let *μ*_i_ denote the mean reward of the i-th arm (strategy). We use a prior distribution on *μ* as p(*μ*_*i*_) = ${\cal{N}}\left( {m_0,{\mathrm{s}}_0} \right)$, where we select *m*_0_ = 0 for each strategy to start with a prior belief that each strategy is the baseline strategy. The standard deviation s_0_ is set to 0.3 to cover the possibilities of mean reward *μ*_*i*_ to lie between [−1, 1] with high probability (greater than 0.999). We also assume the reward observation model to be Gaussian distributed as $r_{i,c,\tau } = {\upmu}_i + \epsilon _{{\mathrm{c}},{\uptau}}$, where ${\it{\epsilon }}_{c,\tau }$ is an i.i.d. Gaussian random variable with zero mean and standard deviation 1. The data collected from all the clinics up to week *t* is denoted as $D_t = \left\{ {i(c,\tau ),r_{i\left( {c,\tau } \right),c,\tau }} \right\}_{\tau = 1}^t$ for all *c*, where $i(c,\tau )$ denotes the strategy used in week *τ* at clinic *c*. After observing data *D*_*t*_, the posterior distribution of *μ*_i_ is given as $p\left( {\mu _i{\mathrm{|}}D_t} \right) = {\cal{N}}\left( {m_t,{\mathrm{s}}_t} \right)$ with mean *m*_*t*_ and standard deviation s_*t*_ given as (see Eq. (3)):

3 $$m_t = s_t\left[ {{\sum \limits_{c = 1}^C} {\sum\limits_{\tau = 1}^t} r_{i(c,\tau ),c,\tau } + \frac{{m_0}}{{s_0^2}}} \right],\,\frac{1}{{{\mathrm{s}}_t}} = \sqrt {tC + \frac{1}{{s_0^2}}}$$

Using the posterior distribution on *μ*_*i*_ we draw a random sample of *μ*_*i*_’s for all arms and then play the arm with the highest *μ*_i_. We repeat this procedure C times to select one strategy for each clinic, a scheme known as parallel Thompson sampling. It has been shown theoretically that such a scheme will converge to the best strategy or arm at an optimal rate.^30^

A one-sided paired *t*-test was used to test the hypothesis of whether a particular strategy performed better than baseline for each GP. The samples used in hypothesis testing are GP-Patient PA discussion scores. These scores are mutually independent for a strategy both:

- Across GPs in any clinic because each GP acts independently, and
- Across weeks each time this strategy was implemented because although strategy selection is dependent on the historical selection of other strategies, previously selected strategies have no bearing on the observed reward from a selected strategy. Rather, the reward is only dependent on the strategy chosen, the GP trialling this strategy, and the patients visiting the GP during the week.

Since the samples are independent, there is no implication on the size of the test and therefore these is no expectation of an increase in false positives (i.e. Type-1 errors).

Due to the multiple hypothesis tests undertaken we applied the Bonferroni correction for a test statistic *p* = 0.05 for the overall multiple hypothesis test. Across eight trials the correction gives *p* = 0.05/8 = 0.006 for each binary test of whether a strategy is better than baseline.

### Ethical approval

Full ethics approvals have been received for all methods described above: DeakinUniversity’s Human Ethics Advisory Group (DU-HEAG) reference HEAG-H 181_2018. Informed written consent was obtained from all human participants in this study.

### Reporting summary

Further information on experimental design is available in the Nature Research Reporting Summary linked to this paper.

## Supplementary information

Supplementary files

Reporting summary

## Data Availability

The anonymised data that support this study are available publicly at https://deakin365-my.sharepoint.com/:f:/g/personal/stephan_jacobs_deakin_edu_au/EhOCNCE_wpNCphnyuXB-hFgBm8Y5QYDfL8JEYFFDuQDVqg?e=rCN6GD.

## Code availability

The code replicating key figures and tables are available publicly at https://deakin365-my.sharepoint.com/:f:/g/personal/stephan_jacobs_deakin_edu_au/EhOCNCE_wpNCphnyuXB-hFgBm8Y5QYDfL8JEYFFDuQDVqg?e=rCN6GD.
