# Supplementary material for: Bayesian strategy selection identifies optimal solutions to complex problems using an example from GP prescribing
Source: NPJ Digit Med. 2020 Jan 20;3:7. doi: 10.1038/s41746-019-0205-y (PMC6971230; doi:10.1038/s41746-019-0205-y)
Supplement: Supplementary file 1 — Supplementary files [file 41746_2019_205_MOESM1_ESM.pdf]

SUPPLEMENTARY FILES

Supplementary Figure 1 Effect (reward) of strategy on GP patient interactions over time (iteration)

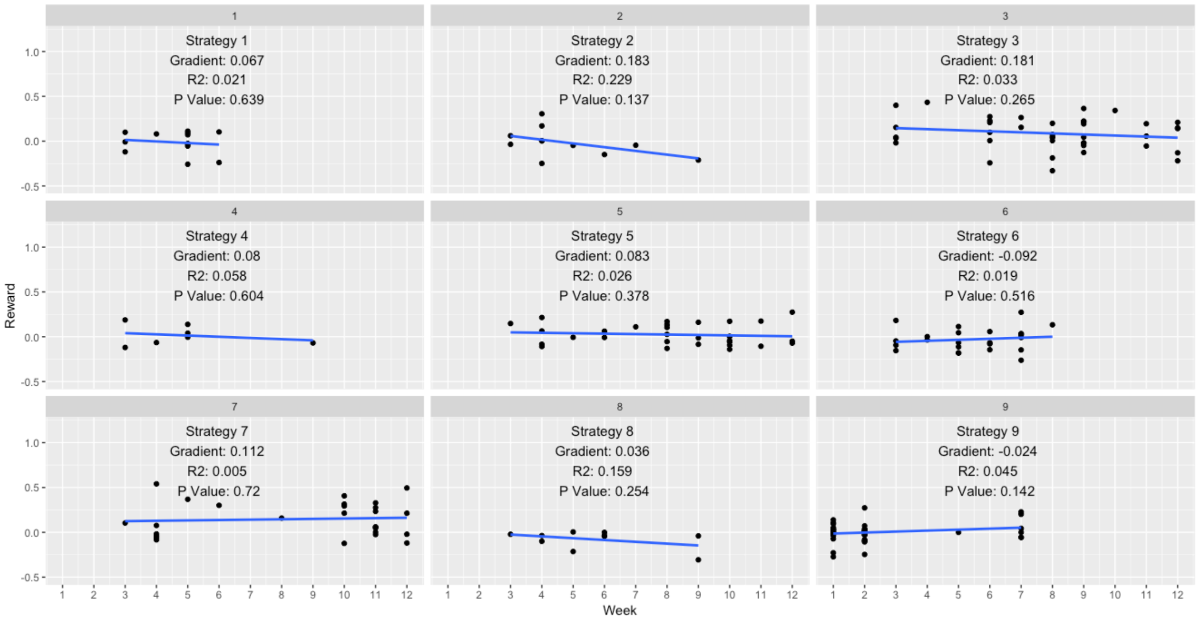

Supplementary Figure 1 shows the observed outcomes for each strategy (all GPs for all clinics) over time with a best fit regression line, estimate for gradient of the line, explained variance (R^2) and hypothesis test to significant gradient (correlation) between time and strategy effect ($P<0.05$). No significant trends exist as seen through low estimated values of gradient, R^2 and all p values >0.05.

Supplementary Figure 2: Adjusted Rand Index for correlation between GP

We take the average data for a GP for each strategy over whole study period), and construct a feature vector. These vectors are then clustered using K-means algorithm (K=No. of clinics) to find correlated groups of GPs based on their score similarity. The idea is to find if such data driven clustering can capture their clinic associations. Using the clinic association of a GP as ground truth, we evaluate the K-means performance using Adjusted Rand Index (ARI).

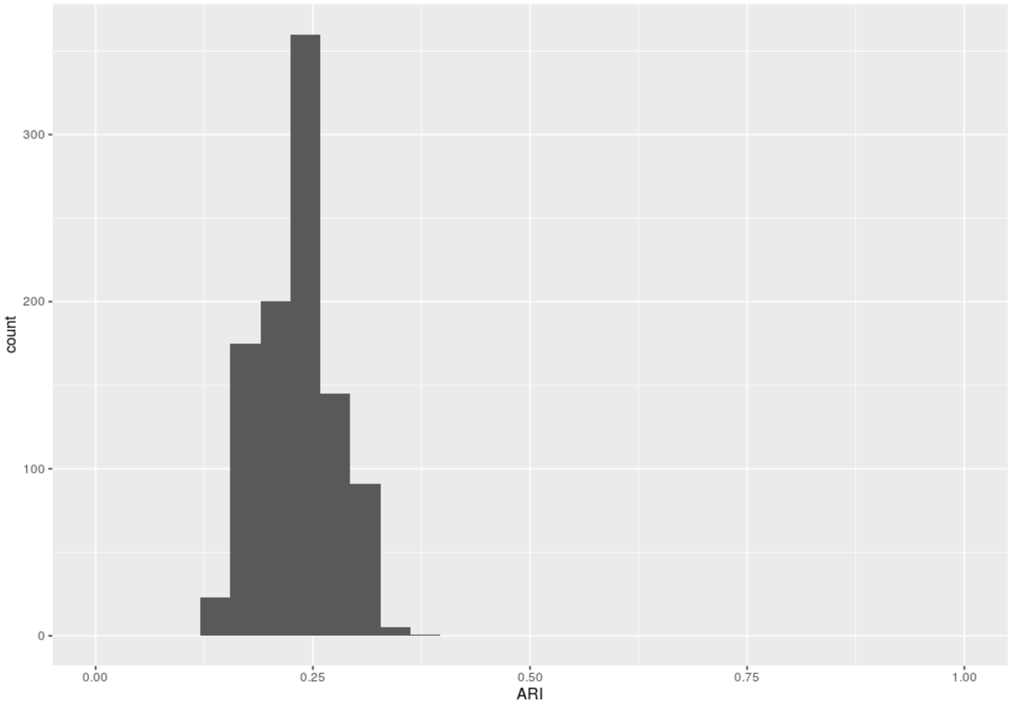

The ARI[1], is routinely used to evaluate clustering performance scores. ARI was chosen because it adjusts for chance allocation to the correct cluster.

Supplementary figure 2 shows ARI histogram for 1000 trials where in each trial K-means is initialised randomly. A weak correlation (mean ARI = 0.23 on the scale of [0,1]) was observed between GPs which indicates independence across GPs.

1. W. M. Rand (1971). "Objective criteria for the evaluation of clustering methods". Journal of the American Statistical Association. American Statistical Association. 66 (336): 846–850. arXiv:1704.01036. doi:10.2307/2284239. JSTOR 2284239.

Supplementary Table 1 Hypothesis test results for all strategies at the end of Stage-2

Strategy number	Mean difference (strategy-baseline)	P Value	Trial Count
7	0.135	0.036	10
3	0.090	0.005	30
5	0.039	0.068	18
4	0.015	0.368	7
1	-0.014	0.651	13
2	-0.018	0.640	11
6	-0.029	0.863	24
8	-0.080	0.982	10

One sided t-test comparing estimates for mean estimate of difference in GP/ patient consultations on PA at the end of Stage-2

Sorted by descending difference in mean effects of strategy and baseline. The top-3 strategies (Strategies 7, 3 and 5) were allowed to progress to Stage-3 for additional trial.
